# Human milk banking can be an innovative approach for developing countries 

**DOI:** 10.15171/hpp.2018.34

**Published:** 2018-10-27

**Authors:** Yasmin Jahan, Atiqur Rahman

**Affiliations:** ^1^Graduate School of Biomedical & Health Sciences, Hiroshima University, Hiroshima, Japan; ^2^School of Health, University of New England, Armidale, Australia

## Background stating the problem

Breast milk is fundamental to the improvement of the infant’s immature vulnerable framework and decreases child mortality. The neonates who are not able to breast feed sustain prematurity after birth and at most danger of not receiving human milk.^1^ Mother’s own milk should the first choice for all neonates including preterm newborn children, when a mother’s own milk is inaccessible or hard to find for different reasons, the following best choice is donated human milk (DHM) as a protected option following World Health Organization (WHO).^2^ The utilization of donor human milk (HM) is expanding for high-risk babies, principally for newborn children conceived measuring <1500 g or those who have serious intestinal issues.^3^ In preterm newborn children, there are particular benefits identified with HM that diminishes fundamentally the danger of stomach related intolerance, necrotizing enterocolitis, late-onset sepsis, bronchopulmonary dysplasia, and retinopathy of prematurity. It has likewise a long-haul positive effect on cognitive development and metabolism and cardiovascular wellbeing at grown-up age.^4^ Along these lines, it is expected that better to give mother’s own milk if impractical at that point give DHM or milk sharing rather than HM substitute.^5^

The rate of exclusive breastfeeding, which makes no difference aside from HM until a half year of age, is 55% in Bangladesh, as per 2014 Demographic and Health Survey (BDHS), yet at the same time, 45% stays out of this which surge newborn child death rates.^6^ As indicated by the World Bank collection of development indicators, in Egypt EBF was accounted for at 39.7% in 2014^7^ and in Indonesia, 42% EBF practices works on, as indicated by the 2012 Indonesian Demographic Health Survey report.^8^ Not just that yet all around just 36% of babies younger than a half year are solely breastfed, and in developing countries, poor feeding practices including lack of exclusive breastfeeding until the point when a half year and inability to start breastfeeding in the first hour which contribute to the deaths of 800 000 children under 5 years old every year.^9^

Furthermore, nurturing an infant in all-day occupation can challenge anyplace in the world. However, in Bangladesh, the rights mounted to work mothers lag way behind. There are around 3.6 million ladies employed in the ready mate garments industry alone, making it the single biggest employer of women in the nation.^10^ These mothers need to work extend periods of time, and numerous industrial facilities do not have creche facilities for young children to empower mothers to convey their kids to work. In this circumstance, they can stop or offer the surplus milk for their child or other people who can utilize it.

Regardless of its significance, HM sharing is still negligibly explored in the scientific literature. A very few researches, researchers have accentuated the requirement for more research on HM sharing that features donor’s demographic profiles and their inspirations, sentiments, mentalities, and convictions concerning this practice.^11^ In perspective of the different psychosocial factors associated with HM sharing, the present study meant to motivate the milk donors about milk sharing.

## Presentation and strategy

Mothers milk is the ideal nutrition for human newborn child nourishment; however, seldomly it is not generally accessible. The utilization of shared HM is not mostly acknowledged in many developing countries. Nevertheless, HM sharing is influenced by biological variables such socio-social qualities as age, education, political perspectives, financial status, and culture. In a few studies, religious convictions, monetary variables, absence of trust in serological tests, inability to know donors, the probability for mixing up donors, considering methods as unhygienic, absence of data about milk sharing and staff shortage have been appeared to effectively affect milk sharing and acknowledgment of donation.^12,13^ Perspectives about HM donation differ with societies and religions. A research demonstrated that the rate of the women considering HM donation inadmissible for religious reasons was 7.25 times higher.^14^ Nonetheless, HM donation isn’t prohibited by Christianity, Buddhism, and Hinduism. Truth be told, it is supported. Islam has additionally been accounted for to underscore the significance of sharing HM and breastfeeding.^15^ In developing country like Bangladesh, however, there is exclusive breast-feeding counselling program yet there is no counseling program with respect to milk sharing. Where young or under-educated mothers do not certainly learn about the advantages of exclusive breastfeeding. When they find that feeding their infant’s solid food or formula before a half year of age can put the child in danger of illnesses like a diarrheal ailment, mothers are frequently anxious to decide on encouraging just breast milk.^16^ On this respect, if the mother cannot generate milk or whatever other reasons that the mother could not have the capacity to feed her offspring of her own breast milk, that time she can utilize shared HM.

Along with these outlines, on that premise motivational meeting as well as awareness development can be an extraordinary comparison to other approach to change their idea on HM sharing. Motivational interviewing (MI) is a client focused interpersonal skill approach, a generally new intervention in the medicinal services setting that seems promising.^17^ This approach deals with connecting with the client’s inherent inspiration to change and perceives that clients will be at different phases of motivational and behavioral status (e.g., precontemplation, contemplation, preparation, action, and maintenance).^17^ It is cost-effective and there are no special requirements needed to do this. Any health care providers can take training and can do this. It will be feasible for developing courtiers and in low recourse settings. As there are no evidence to do such kind of research in developing countries.

A group of certified MI health care providers will direct visit the women’s including pregnant and lactating mothers. The motivational interviewing advising should be possible in community, clinic, essential health care services adjusting places. The prepared health care providers will direct an advising session on women’s participant and tested their knowledge, perception, and attitude towards HM sharing.

Additionally, the potential milk donors can be paid or unpaid volunteers, so they initially ought to be notable concerning screening methodology for medication use, nutrition status, smoking, alcohol intake, general health status, international travel history, place of living, disease history, substantial risk behavior, history of risks for infectious diseases, state of nipples and the minimum quantity of milk liable to be obtained.^18^

Developing country stay behind whatever remains of the world in building up and advancing HM banks. Moreover, we can similarly educate them how to collect and pasteurized the shared milk for later utilize. With this thought we can spur them for creating milk bank for different infants who required milk by paid or unpaid volunteers.

## Conclusion

It can be prescribed that endeavors to illuminate about distinguish the elements influencing HM donation and to bring issues to light about the issue ought to be made and that essential directions for HM banks should be embraced. Women’s ought to be taught about the decreased danger of irresistible ailment with pasteurized HM. Additionally, learns about the issue should concentrate on more enumerated studies of misguided recognitions liable to be detrimental regarding HM sharing. Authorities ought to coordinate with the pioneers to accomplish more uplifting states of awareness towards HM donation as well as sharing and HM banking as a safe and financially savvy method.

Taking everything into account, all nations need to incorporate donor HM managing an account as a major aspect of the aggregate maternal and child wellbeing strategy, with the goal that it is done firmly and reliably and is open to infants and children in require.^19^ To build up this approach, most extreme exertion ought to dependably be placed in supporting and advancing milk donation and milk banking as a contrasting option to mother’s milk as well as a breastfeeding advancement and HM provision strategy. In supporting donation HM, we will really be cultivating a child neighborly world.

## Ethical approval

Not Applicable.

## Competing interests

The authors declare that they have no competing interests.

## Authors’ contributions

YJ conceived the study, collected the data, conducted the analysis and interpret the data and wrote the manuscript. AR oversaw the whole study process, designed the study, analyzed and interpreted data, and critically reviewed the manuscript. All authors read and approved the final manuscript.
